# p53 regulates the transcription of the anti-inflammatory molecule developmental endothelial locus-1 (Del-1)

**DOI:** 10.18632/oncotarget.1318

**Published:** 2013-10-03

**Authors:** Hyesoon Kim, Seung-Hwan Lee, Mi-Ni Lee, Goo Taeg Oh, Kyung-Chul Choi, Eun Young Choi

**Affiliations:** ^1^ Department of Biomedical Sciences, University of Ulsan College of Medicine, Seoul, Republic of Korea; ^2^ Division of Life Sciences, Ewha Womans University, Seoul, Republic of Korea; ^3^ Department of Pharmacology, University of Ulsan College of Medicine, Seoul, Republic of Korea

**Keywords:** p53, inflammation, endothelial cell, Del-1

## Abstract

Developmental endothelial locus-1 (Del-1) is an endothelium-derived anti-inflammatory molecule that is downregulated by inflammatory stimuli. Little is known about the molecular mechanisms by which Del-1 transcription is regulated. In the present study, a DNA sequence upstream of the Del-1 gene was analyzed and putative p53 response elements (p53REs) were identified. An approximately 2 kb fragment upstream of the translation start site displayed the highest Del-1 transcriptional activity, and the transcriptional activity of this fragment was enhanced by overexpression of p53. Chemical activation of endogenous p53 elevated the levels of Del-1 mRNA. Site-directed mutagenesis of CATG in the consensus sequences of the 2 kb fragment to TATA significantly reduced the transcription of Del-1. Chromatin immunoprecipitation revealed recruitment of p53 to the p53REs of the Del-1 promoter, resulting in increased Del-1 transcription. Finally, primary endothelial cells isolated from mice with reduced levels of p53 showed a decrease in Del-1 mRNA compared to wild-type endothelial cells. Moreover, Del-1 reciprocally enhanced p53 expression in primary endothelial cells. Thus, these findings suggest that Del-1 is a novel transcriptional target gene of p53.

## INTRODUCTION

Developmental endothelial locus-1 (Del-1, also called Edil3) is a 52 kD glycoprotein that harbors three epidermal growth factor-like domains and two discoidin domains. Although Del-1is secreted by endothelial cells, it can be associated with proteoglycans on the cell surface or can bind to endothelial integrins such as α_v_β_3_ and α_v_β_5_. Del-1 has been proposed to be implicated in the vascularization, angiogenesis, apoptosis, adhesion, migration, and proliferation, although the involvement of Del-1 in these cellular activities remains controversial [1-5]. We previously reported that Del-1 competes with the endothelial cell adhesion molecule ICAM-1 for binding to leukocyte function-associated antigen 1 (LFA-1), thereby inhibiting leukocyte adhesion and subsequent migration through the vessel barrier. By limiting leukocyte recruitment, Del-1 inhibits the duration and magnitude of the inflammatory response [6].

Del-1 expression is tissue-specific in mice. Del-1 is highly expressed in the lung and central nervous system, but there is little or no expression in other tissues, suggesting that tissue-specific transcription factors are involved in the expression of Del-1 in mice [6]. Human Del-1 is more than 97% homologous to its murine counterpart. However, bioinformatic studies revealed that human Del-1 is almost ubiquitously expressed, suggesting the presence of a universal modulator for Del-1 expression in humans. We previously demonstrated that Del-1 is expressed in endothelial cells, in which Del-1 expression is downregulated by inflammatory stimuli such as tumor necrosis factor (TNF)-α, lipopolysaccharide (LPS), and interleukin (IL)-17 [6, 7]. With age, the expression of Del-1 decreases while IL-17 expression increases, and Del-1-deficient mice experience spontaneous inflammatory bone loss, signifying that Del-1 is involved in the maintenance of tissue homeostasis against inflammatory pathology [8, 9].

Our previous study demonstrating that Del-1 is downregulated in response to inflammatory stimuli raises the possibility that NF-κB activation might control Del-1 transcription. Conversely, it also implies that inhibition of NF-κB may lead to the upregulation of Del-1. A search for possible modulators of Del-1 expression led to the observation that histone deacetylase (HDAC) inhibitors such as valproic acid and butyric acid increased Del-1 mRNA (data not published). Valproic acid and butyric acid are known to inhibit the NF-κB pathway and to activate p53 [10-12]. These findings suggest that there might be factors that enhance Del-1 production while counteracting NF-κB. Given the interaction between NF-κB and p53 [13, 14], and that chronic inflammation (i.e., continuous NF-κB activation) is associated with cancer development (i.e., through p53 inactivation), we investigated a potential link between the anti-inflammatory molecule Del-1 and the tumor suppressor p53.

As Del-1 inhibits leukocyte recruitment, Del-1 is a strong candidate for therapeutics that target inflammatory pathways. Hence, any molecule that modulates the expression of Del-1 may be of therapeutic value. However, little is known about the molecule to date. Here, we report that upstream region of the Del-1 gene harbors multiple p53 response elements, mutation of which decreased Del-1 transcriptional activity. Del-1 transcription was upregulated by active p53 and mediated by direct binding of p53 to the upstream fragment of the Del-1 gene. In addition, primary endothelial cells containing reduced levels of p53 had decreased Del-1 expression, indicating a significant role for p53 in the regulation of Del-1 expression to maintain tissue homeostasis.

## RESULTS

### Identification of p53 response elements upstream of the Del-1 gene

**Figure 1:**
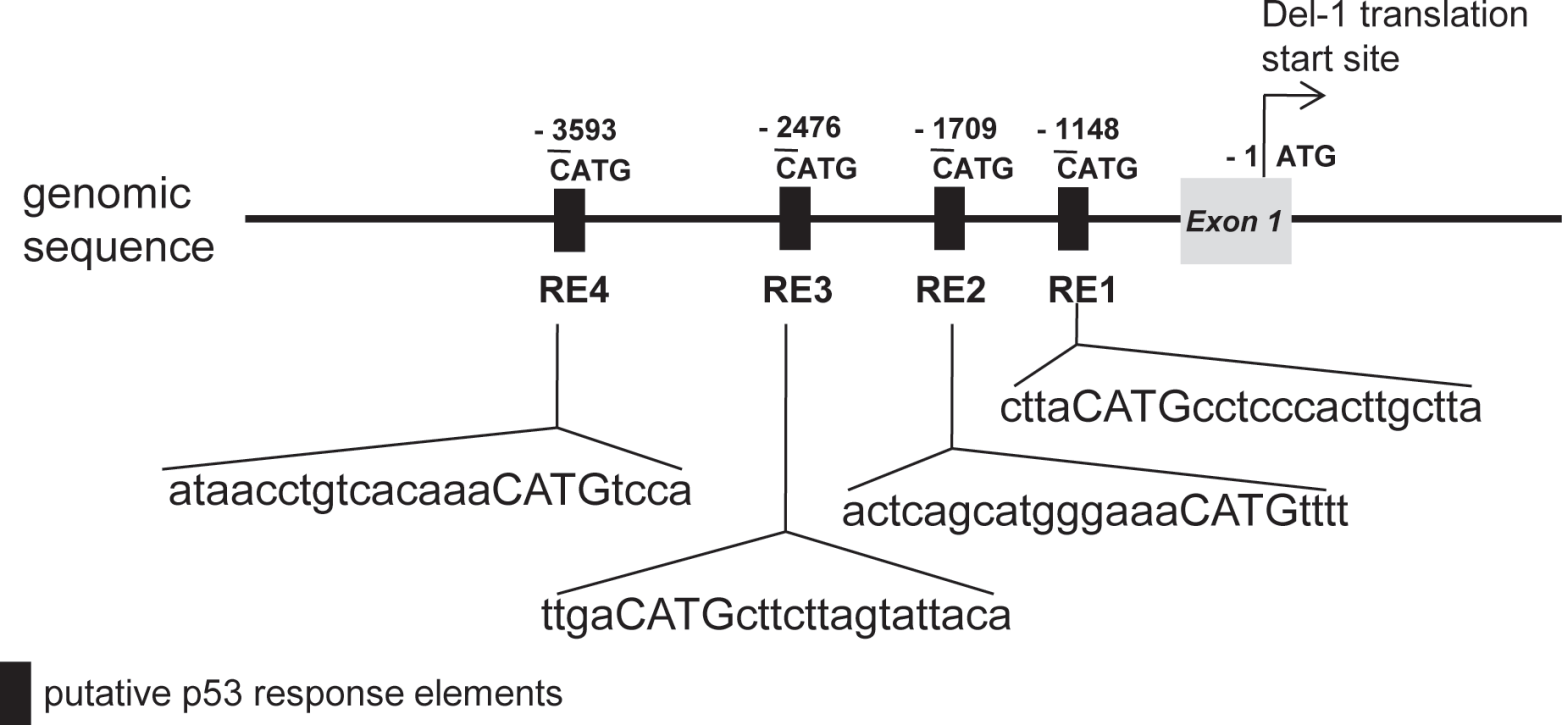
Schematic representation of the region upstream of the Del-1 gene Putative p53 response elements (p53REs) were predicted using the Genometrix program. CATG-containing p53REs in the upstream regions are indicated. The first nucleotide of CATG is numbered relative to the translation start site (TSS) of the Del-1 gene.

**Figure 2:**
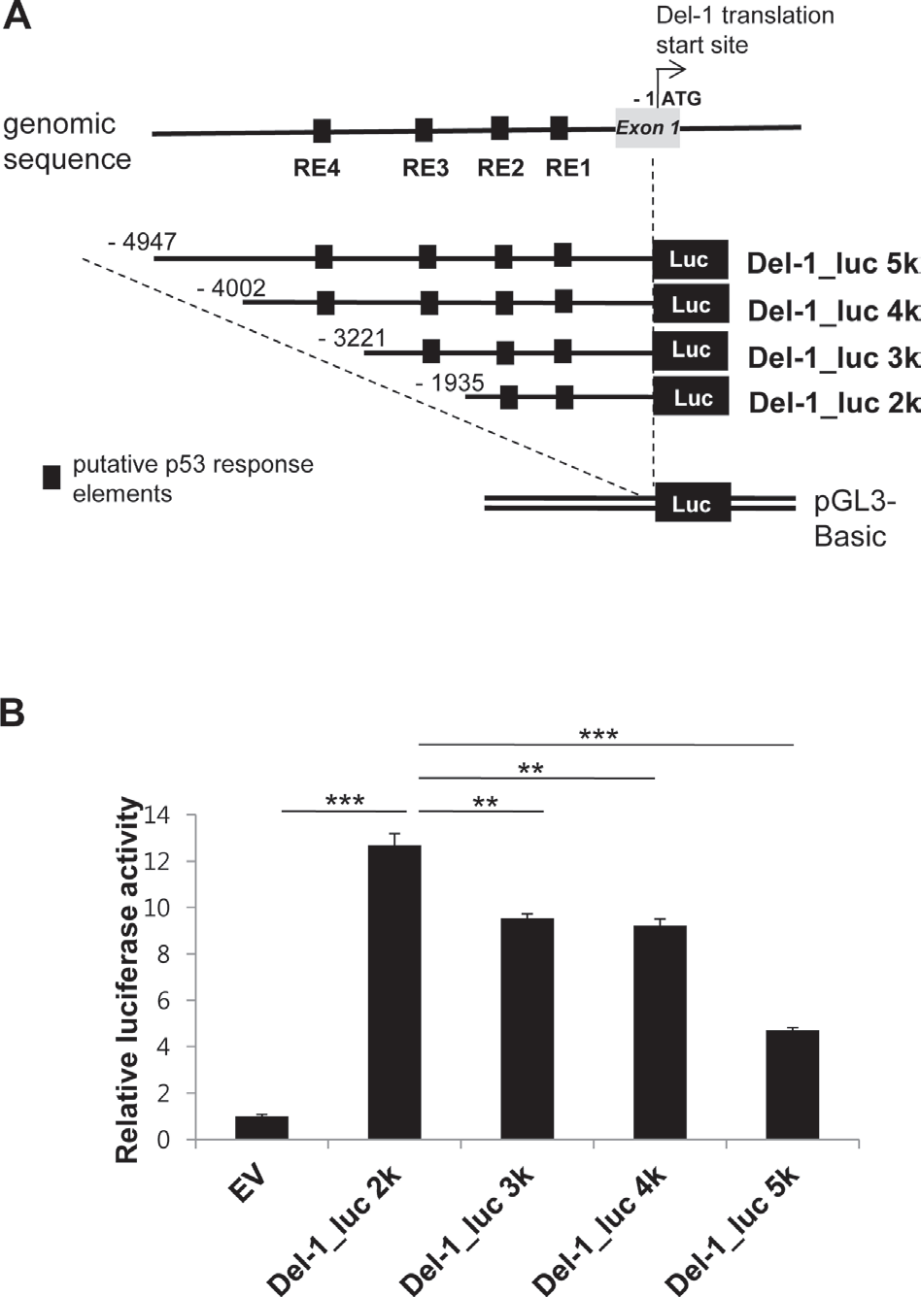
Relative transcriptional activity is dependent on the presence of proximal p53 response elements in the upstream region of the Del-1 gene (A) Schematic diagram of the Del-1 promoter constructs. Nucleotides are indicated relative to the TSS. Upstream fragments of the Del-1 gene were cloned into the pGL3 vector to generate four Del-1 promoter deletion constructs containing multiple putative p53 response elements. (B) Relative luciferase activity of these constructs. The Del-1 promoter constructs shown in (A) were individually transfected into HEK293T cells. Luciferase activity was determined 24 h after transfection and is expressed as the fold activity over that of the empty pGL3 vector (EV). A *Renilla* luciferase vector was co-transfected for normalization of transfection efficiency. Values are the means ± standard deviations (SD) from triplicate transfections. Data represent four independent experiments. **, *p* < 0.01; ***, *p* < 0.001; *n.s.*, non-significant vs. the Del-1_luc 2k construct (i.e., the construct exhibiting the highest activity).

**Figure 3:**
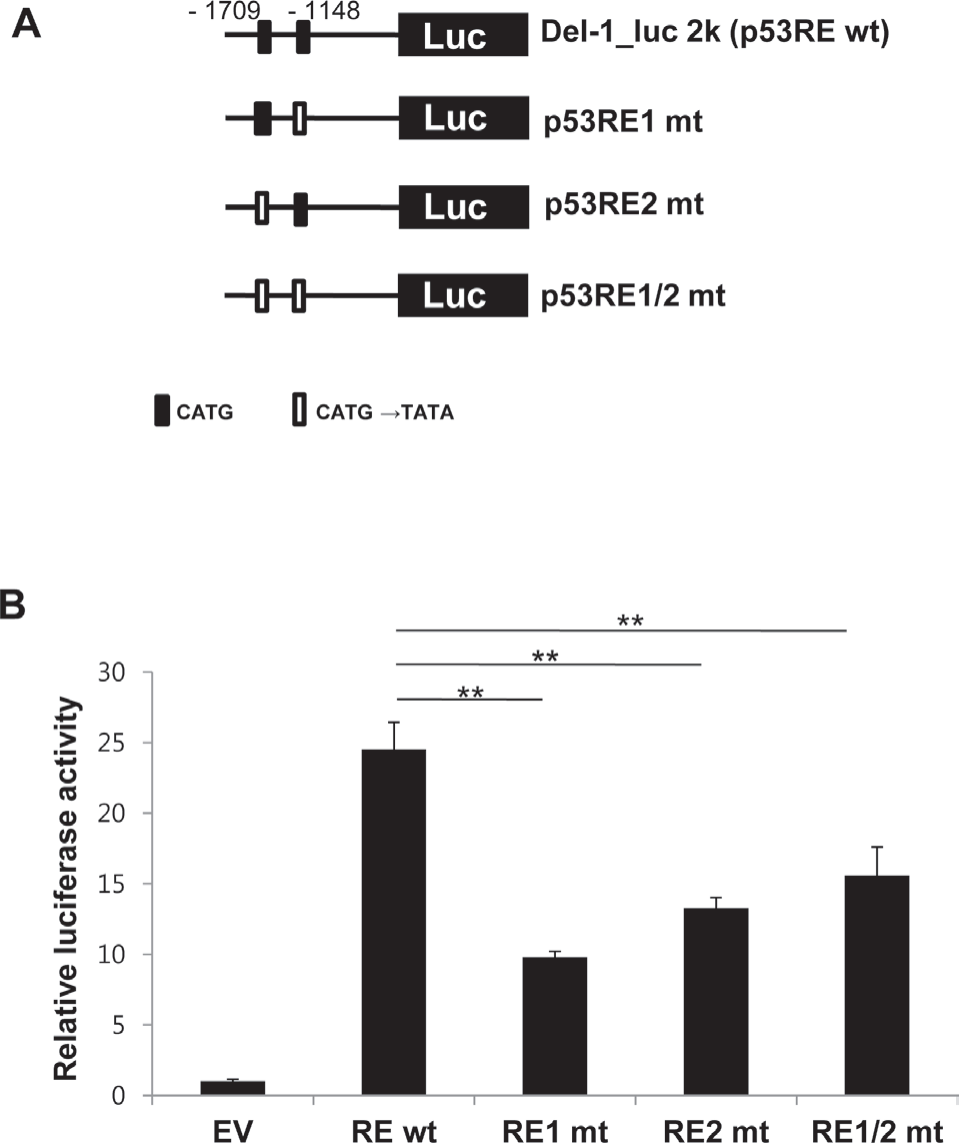
Functional p53 response elements are required to enhance Del-1 transcription (A) Schematic diagram of wild-type (WT) and mutant Del-1 promoter constructs. The Del-1_luc 2k construct was mutated at the consensus sequence (CATG→TATA) of either or both p53 response elements. (B) The WT and mutant Del-1_luc 2k constructs were independently transfected into HEK293T cells. Luciferase activity was determined 24 h after transfection and is expressed as the fold activity over that of the empty pGL3 vector (EV). Values are means ± SD from triplicate transfections. Data represent four independent experiments. *, *p* < 0.05; **, *p* < 0.01; *n.s.*, non-significant vs. the WT Del-1_luc 2k construct.

A search for the upstream region of the Del-1 gene using Matlnspector (Genomatix; http://www.genomatix.de) revealed four putative p53 response elements (p53REs) (Fig. 1). To determine if these elements are functional, four constructs with various lengths (2–5 kb) of the region upstream of the Del-1 translation start site (TSS) fused to the luciferase gene (Fig. 2A) were generated. These constructs were transfected into HEK293T cells and luciferase activity was assessed. The construct harboring an approximately 2 kb fragment upstream of the Del-1 TSS (Del-1_luc 2k) elicited the highest luciferase activity (Fig. 2B) and was therefore used in subsequent analyses. As the 2 kb fragment harbors two putative p53REs, it was hypothesized that these p53REs might be involved in the regulation of Del-1 transcription. The p53REs of Del-1 are non-canonical REs. While canonical REs are composed of two 10-base decamers (RRRCWWGYYY, in which R is a purine and Y is a pyrimidine) and a spacer of 0–13 nucleotides between the two decamers, non-canonical REs include half and three-quarter sites, which are also functional [15]. To determine if the p53REs are functional for Del-1 transcription, CATG in the consensus sequences of each of the p53REs in the 2 kb fragment were mutated alone or in combination to TATA. A luciferase assay revealed that mutation of either p53RE significantly reduced the transcriptional activity (Fig. 3), demonstrating an important role for these p53REs in the transcriptional regulation of Del-1. Of note, mutation of CATG in both p53RE did not reduce the luciferase activity further than that in a single p53RE, suggesting that either of p53REs, but not both, is required for the transcription of Del-1.

### p53 activation up-regulates Del-1 transcription

**Figure 4:**
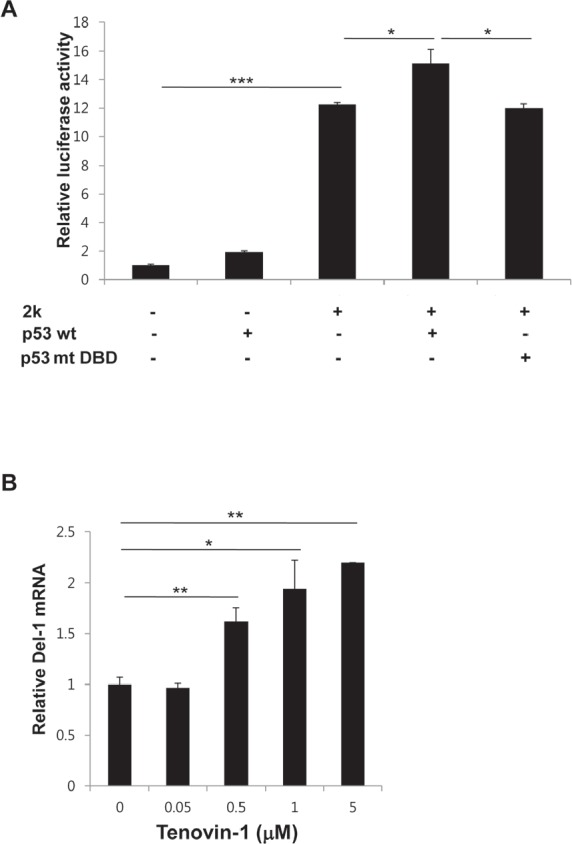
p53 positively regulates Del-1 transcription (A) Mouse WT p53 or p53 with mutated DNA binding sites (G239A, R242A, and R243A) was transfected along with the 2k Del-1 promoter construct into HEK293T cells. Luciferase activity was determined 24 h after transfection and is expressed as the fold activity over that of the empty pGL3 vector. Values are means ± standard deviations from triplicate transfections. *, *p* < 0.05; ***, *p* < 0.001, vs. the 2k Del-1 promoter construct. (B) Mouse primary endothelial cells were treated with increasing concentrations of tenovin-1 and incubated for 24 h. The Del-1 mRNA level was measured and is expressed as the fold increase over dimethyl sulfoxide (DMSO)-treated cells. Values are means ± SD from triplicate treatments. Data represent three independent experiments. *, *p* < 0.05; **, *p* < 0.01 vs. the DMSO-treated cells.

p53 binds to p53RE and increases transcription of downstream genes [16, 17]. To determine if binding of p53 to the p53REs induces transcription of the Del-1 gene, Del-1 transcriptional activity was evaluated following p53 overexpression. A mouse p53 allele was generated containing mutations in Gly-239, Arg-242, and Arg-243 of the DNA binding domain (DBD). These three amino acid residues correspond to Gly-245, Arg-248, and Arg-249 in the DBD of human p53, mutations of which frequently lead to cancer development [18, 19]. HEK293T cells were transfected with the Del-1_luc 2k along with plasmids encoding wild-type (WT) or mutant p53. Indeed, co-transfection of WT p53, but not p53 containing the DBD mutations, significantly increased luciferase activity (Fig. 4A), indicating that p53 directly binds to the Del-1 upstream fragment, inducing Del-1 expression. To investigate p53-mediated upregulation of Del-1 in a more endogenous setting, we turned to tenovin-1, an inhibitor of the interaction between p53 and MDM2 [20]. As MDM2 is an E3 ubiquitin ligase that downregulates p53, tenovin-1 treatment results in p53 activation. Endothelial cells isolated from mouse lungs were treated with tenovin-1 and processed for real-time RT-PCR. Tenovin-1 raised Del-1 mRNA levels compared to control-treated cells in a dose-dependent manner (Fig. 4B), demonstrating that chemical activation of p53 in endothelial cells also enhances Del-1 transcription. Taken together, these findings suggest that p53 plays an important role in the transcriptional regulation of Del-1.

### p53 binds to p53REs in the Del-1 promoter

**Figure 5:**
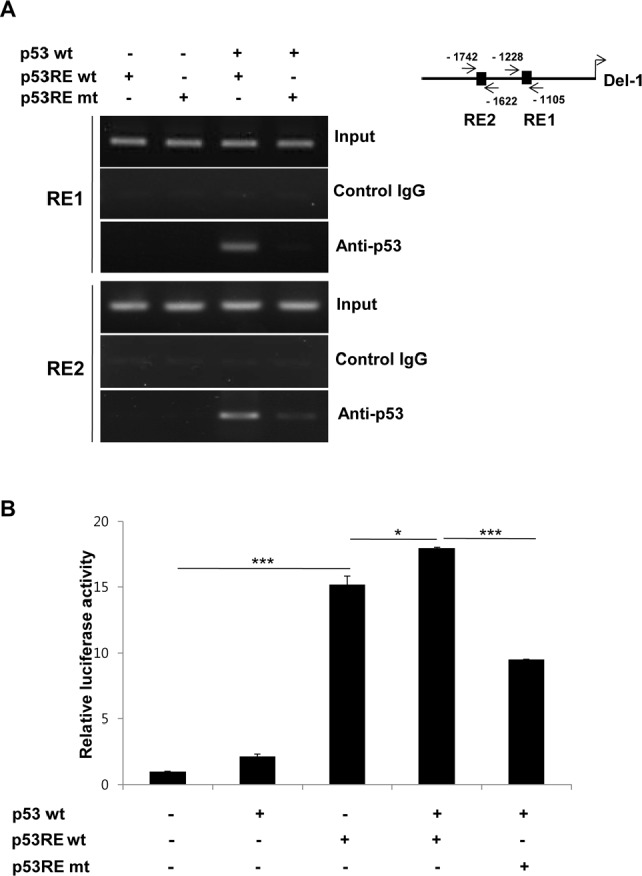
p53 directly binds to p53 response elements to enhance Del-1 transcription (A) Representative ChIP analysis. HEK293T cells were transfected with a mouse p53-expressing plasmid, together with either the WT or mutant (mutated at both p53REs) 2k Del-1 promoter construct. Nuclear lysates were analyzed 48 h after transfection using a p53 antibody or control IgG and promoter regions containing the p53REs were amplified by PCR. (B) HEK293T cells were transfected with the plasmids in (A). Luciferase activity was determined 24 h after transfection and is expressed as the fold activity over that of the empty pGL3 vector. Values are means ± SD from triplicate transfections. Data represent three independent experiments. *, *p* < 0.05; ***, *p* < 0.001 *vs.* the cells co-transfected with the WT p53 and p53REwt Del-1 constructs.

To determine whether p53 directly binds to the p53REs in the Del-1 promoter, HEK293T cells were transfected with a p53 expression plasmid and a Del-1_luc 2k construct containing WT or mutated p53REs, and chromatin immunoprecipitation was performed using a p53 antibody. p53 bound to the 2 kb fragment containing the WT p53REs, but did not bind to the 2 kb fragment containing mutant p53REs (Fig. 5A), suggesting that p53 directly binds to the 2 kb fragment upstream of the Del-1 gene via the p53REs. The dependence of Del-1 transcription on the interaction between p53 and the p53REs was next examined. Indeed, transfection of HEK293T cells with WT p53 plus a construct consisting of the WT p53REs fused to the luciferase gene resulted in higher Del-1 luciferase activity than WT p53 plus a construct containing mutant p53REs (Fig. 5B), suggesting that the upregulation of Del-1 transcription by p53 is dependent on the p53REs.

### p53 deficiency reduces Del-1 expression in mouse primary endothelial cells

**Figure 6:**
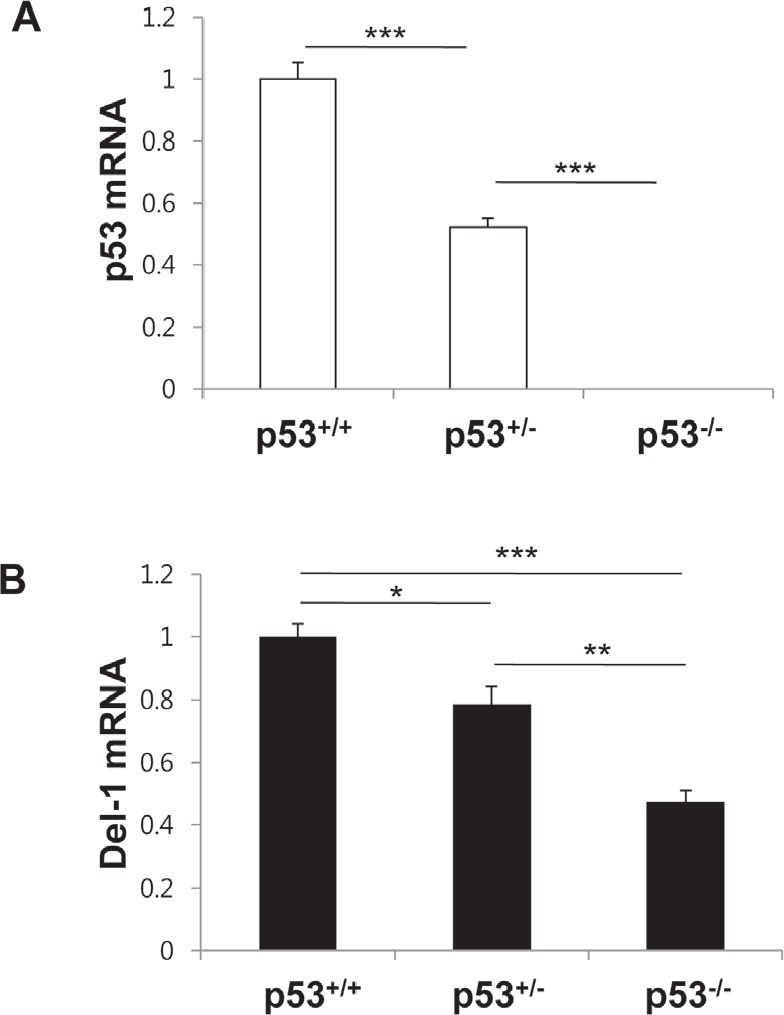
p53 levels determine Del-1 expression in mouse primary endothelial cells Mouse p53 (A) and Del-1(B) mRNA levels were analyzed by real-time RT-PCR in primary endothelial cells from p53^+/+^, p53^+/−^, and p53^−/−^ mice. The relative Del-1 mRNA levels in cells heterozygous or homozygous null for p53 were compared to those of p53^+/+^ mice. Values are means ± SD (n = 4–5 mice/group). Data represent three independent experiments. *, *p* < 0.05; **, *p* < 0.01; ***, *p* < 0.001.

To evaluate p53-mediated induction of Del-1 expression in an *in vivo* setting, lung endothelial cells were isolated from WT mice (p53^+/+^) or mice homozygous (p53^−/−^) or heterozygous (p53^+/−^) null for p53, and p53 and Del-1 mRNA levels were assessed by quantitative real-time PCR. As expected, p53 levels were undetectable or reduced in p53^−/−^ and heterozygous p53^+/−^ cells, respectively (Fig. 6A). Consistent with the *in vitro* data, p53^−/−^ cells displayed decreased Del-1 mRNA levels compared to WT cells. Of note, levels of Del-1 mRNA in the p53^+/−^ cells were less than those in p53^+/+^ cells but greater than those in p53^−/−^ cells (Fig. 6B), indicating that the Del-1 expression level correlates with the amount of p53 in primary endothelial cells.

### Del-1 reciprocally regulates p53 expression in mouse primary endothelial cells

**Figure 7:**
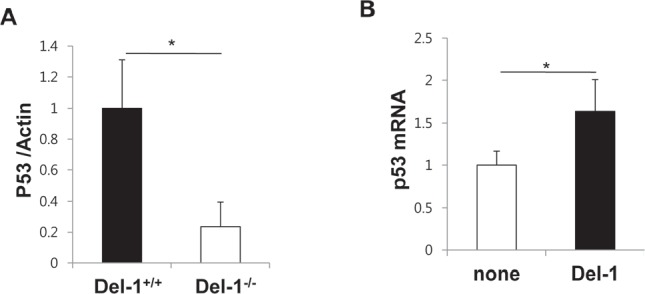
Del-1 reciprocally regulates p53 expression in mouse primary endothelial cells (A) Levels of p53 protein was analyzed by western blotting in primary endothelial cells from Del-1^+/+^ and Del-1^−/−^ mice. Values are means ± SD (n = 3 mice/group). (B) Transcript levels of p53 were analyzed by real-time RT-PCR. WT primary endothelial cells were incubated in the absence or presence of recombinant Del-1 protein (10 nM) for 24 h. Values are means ± SD (n = 4−5 mice/group). *, *p* < 0.05.

As p53 protects cells against a variety of cellular stresses and often acts as a negative regulator of inflammation [13, 21], modulation of p53 function is a promising treatment strategy for p53-associated pathologies. Del-1 functions as an anti-inflammatory molecule, and many anti-inflammatory agents are capable of activating p53 [22, 23]. Having established that p53 regulates Del-1 expression, we therefore next investigated whether Del-1 reciprocally regulates p53 expression and activation. Primary endothelial cells isolated from Del-1^−/−^ mice showed decrease in p53 protein than those from WT mice (Fig. 7A). Furthermore, the addition of Del-1 to WT primary endothelial cells enhanced p53 mRNA expression (Fig. 7B). Taken together, these findings suggest that Del-1 and p53 reciprocally regulate the production of each another.

## DISCUSSION

Here, we show that the DNA region upstream of the Del-1 gene contains multiple p53 response elements (p53REs). Mutation of the p53REs reduced Del-1 transcriptional activity and activation of endogenous p53 promoted Del-1 expression in primary endothelial cells. Recruitment of p53 to the p53REs led to an increase in Del-1 transcription. In addition, p53 deficiency resulted in a decrease in Del-1 mRNA in mouse endothelial cells. Finally, Del-1 and p53 reciprocally regulate each other's expression. To the best of our knowledge, this is the first report to identify Del-1 as a downstream target of p53.

Four deletion constructs were generated containing the Del-1 promoter fused to luciferase. The highest luciferase activity was observed using the 2 kb fragment, which contains two REs. However, the other three fragments (3 kb, 4 kb and 5 kb), elicited lower luciferase activity than the 2 kb fragment although all four fragments contained the two p53REs (Fig. 2B). Of note, the 3 kb fragment contains a total of three REs (the two REs in the 2 kb fragment plus a third RE), and the 4 kb and 5 kb fragments contain four REs (the three REs in the 3 kb fragment plus a fourth RE). Several possibilities, which are not mutually exclusive, may explain this discrepancy. First, the third and fourth REs may not be functional p53 binding sites, which would explain the failure of the 3 kb and 4 kb fragments (containing one or two additional REs) to induce higher luciferase activity than the 2 kb fragment. Second, regulatory regions that suppress the transcription of Del-1 may exist in the 3, 4, and 5 kb fragments. Our data suggests that there are at least two inhibitory regulatory regions. The first may reside between −2 kb (with respect to TSS) and −3 kb, as the luciferase activities of the 3 and 4 kb fragments were comparable and lower than that of the 2 kb fragment. The second may reside between −4 kb and −5 kb, as the luciferase activity of the 5 kb fragment was even lower than that of the 3 and 4 kb fragments. Further study is required to identify and characterize these inhibitory regulatory regions.

Expression of Del-1 is not ubiquitous in mice. Rather, it is limited to several tissues, including the lung and brain [6]. Although p53 is ubiquitously expressed, its target genes are not [13, 24], signifying that p53, along with its cofactors, is involved in the tissue-specific expression of its target genes. It is therefore possible that p53 might control the tissue-specific expression of Del-1. Examination of the tissue distribution of Del-1 in p53 null mice will clarify this issue.

We demonstrate here that Del-1 is a target gene of p53. What is then the potential significance of p53 regulation of Del-1 transcription? Since its cloning in 1983 [25], p53 has been considered to be a master regulator of cancer suppression [26, 27]. Accumulating lines of evidence indicate, however, that p53 has a much broader role than initially thought [21, 26, 28-32]. Hence, p53 is now rather viewed as a “stress sensor” in the cell. For example, p53 is implicated in suppression of epigenetic silencing of many non-coding RNAs [33], suppression of aging [34-36], maintenance of a differentiated cellular state [37], and inhibition of oncogenic metabolism [38]. Of these processes in which p53 is implicated, suppression of aging attracts our attention because one of the most known features of aging is inflammation (including atherosclerosis) [39]. Thus, it is tempting to speculate that p53 exerts its anti-aging effect, at least in part, via upregulation of Del-1, an endogenous anti-inflammatory molecule.

## MATERIALS AND METHODS

### Ethics statement

Animal studies were approved by the Asan Institute for Life Sciences Institutional Animal Care and Use Committee (Project number: 2012-14-032) and by the Ewha Women's University Institutional Animal Care and Use Committee (Project number: IACUC-2010-24-2).

### Cell culture

HEK 293T cells were obtained from the American Type Culture Collection (Manassas, VA, USA) and were grown in Dulbecco's modified Eagle's medium (DMEM) supplemented with 10% fetal bovine serum (FBS) and streptomycin/penicillin. All cell culture reagents were purchased from Invitrogen/Life Technologies (Carlsbad, CA) except where otherwise described.

### Isolation of mouse primary endothelial cells

p53 heterozygous mice (B6.129S2-*Trp53^tm1Tyj^*/J) on a C57BL/6 background were purchased from The Jackson Laboratory. These mice were crossed to generate p53^+/+^ (wild-type), p53^+/−^, and p53^−/−^ offspring. Del-1^−/−^ mice were kindly provided by Prof. T. Chavakis (Dresden University, Germany). Mouse lung endothelial cells were isolated and cultured as previously described [6]. In brief, lungs were removed from mice, minced, and digested with type 1 collagenase (2 mg/ml, Gibco/Life Technologies) at 37°C for 1 h. The cellular digest was filtered through a cell strainer and centrifuged, and then the cells were plated on gelatin-coated dishes containing DMEM/F12 medium (Gibco) supplemented with 20% FBS and 100 μg/ml ECGS (BD Biosciences, San Jose, CA). The floating cells were removed on day 1 and fresh medium was added. On days 5 and 10, purification was performed using sheep anti-rat IgG Dynabeads (Invitrogen) which were precoated with a rat anti-mouse ICAM-2 mAb (3C4). The purity of the isolated endothelial cells was verified by PECAM-1 staining on a Accuri C6 flow cytometer (Accuri Cytometers, Inc., Ann Arbor, MI). For some experiments, the cells were treated with tenovin-1 (Tocris Bioscience, Ellisville, MO) or recombinant Del-1 (R&D Systems, Minneapolis, MN) overnight.

### Plasmid construction

To generate Del-1 luciferase constructs, 2–5 kb DNA region (relative to the TSS) upstream of the mouse Del-1 gene were amplified using KOD HOT Start DNA polymerase (Novagen, Nottingham, UK) from the BAC clone RPCI-24 (Children's Hospital Oakland Research Institute, Oakland, CA). The amplified fragments were cloned into the NheI-BglII sites of the pGL3 basic vector (Promega, Madison, WI). The primers used for PCR and cloning of the Del-1 luciferase constructs are listed in Table 1. The mutagenesis of p53RE plasmids was performed using the 2 kb Del-1 construct as a template. The mutant p53-expressing plasmid was generated using the primers listed in Table 1 and the mouse WT p53 plasmid as a template. The primer sequences for mutagenesis are listed in Table 1.

**TABLE 1:** PCR primer sequences for the construction of expression plasmids

PCR primer	DNA sequences (5'→ 3')
Del-1_luc 5k F_NheI	TAGTCGCTAGCAAAGGTGTGTACTAAGTGGCTG
Del-1_luc 4k F_NheI	TAGTCGCTAGCTACAGTCTTTCTACCTCACAGTC
Del-1_luc 3k F_NheI	TAGTCGCTAGCTCAAGGATGCCAAGACGCTTC
Del-1_luc 2k F_NheI	TAGTCGCTAGCAACTCCTGCCTCACCATTCTAG
Del-1_luc R_BglII	TAGTGAGATCTGATCCCTTCTCACGGACGTGA
Del-1 _luc 2k_mut RE1_F	GAAACAGATGTCCTGCTTATATACCTCCCACTTGCTTATG
Del-1_luc 2k_mut RE1_R	CATAAGCAAGTGGGAGGTATATAAGCAGGACATCTGTTTC
Del-1_luc 2k_mut RE2_F	CTCACTCAGCATGGGAAATATATTTTATTGTGGTTGGTG
Del-1_luc 2k_mut RE2_R	CACCAACCACAATAAAATATATTTCCCATGCTGAGTGAG
Mouse mut p53 _F	GTAATAGCTCCTGCATGGGGGCCATGAACGCCGCACCTATCCTTACCATC
Mouse mut p53 _R	GATGGTAAGGATAGGTGCGGCGTTCATGGCCCCCATGCAGGAGCTATTAC

### Transient transfection and luciferase assay

293T cells were plated at a density of 3 × 10^5^ cells per well in a 12-well plate one day prior to transfection. The cells were transfected with 300 ng of the indicated constructs, together with a *Renilla* luciferase plasmid, using the Effectene transfection reagent (Qiagen, Hilden, Germany) in Opti-MEM (Invitrogen, Carlsbad), according to the manufacturer's protocol. Luciferase activity was measured 24 h post-transfection using a dual luciferase reporter assay system (Promega, Madison, WI) and a Victor X3 luminometer (Perkin Elmer), according to the manufacturer's protocol. Of note, plasmid encoding β-galactosidase was used as a control in the transfection experiments with p53RE mutant plasmids due to promoter interference between plasmid encoding *Renilla* luciferase and p53RE mutant plasmids.

### Chromatin immunoprecipitation (ChIP) assay

293T cells were transfected with the indicated plasmids and grown on 150 mm dishes. The cells were cross-linked by adding 1% formaldehyde for 10 min, treated 125 mM glycine (to quench the formaldehyde) for 5 min, washed with ice-cold PBS, collected, and then centrifuged. After centrifugation, the cells were resuspended in a lysis buffer [5 mM PIPES buffer (KOH; pH 8.0), 8.5 mM KCl, 0.5% NP-40, and protease inhibitors], incubated on ice for 10 min, and centrifuged at 5,000 rpm for 5 min. The nuclei were resuspended in a nuclear lysis buffer [50 mM Tris (pH 8.1), 10 mM EDTA, 1% SDS, and protease inhibitors], incubated on ice for 10 min, and then subjected to sonication (producing chromatin fragments of about 0.5–1 kb) on ice. Debris was cleared by centrifugation at maximum speed for 10 min at 4°C. The supernatant containing the chromatin was diluted 1:5 in a ChIP dilution buffer [0.01% SDS, 1.1% Triton X-100, 1.2 mM EDTA, 10.7 mM Tris (pH 8.1), 167 mM NaCl, and protease inhibitors]. The chromatin solution was precleared with protein A/G agarose beads (Santa Cruz Biotechnology, Santa Cruz, CA), immunoprecipitated with p53 antibody (clone DO-1, Santa Cruz) for 5 h at 4°C with rotation, and then combined with protein A/G agarose beads. The beds were washed consecutively with low-salt wash buffer [0.1% SDS, 1% Triton X-100, 2 mM EDTA, 20 mM Tris (pH 8.1), and 150 mM NaCl], high-salt wash buffer [0.1% SDS, 1% Triton X-100, 2 mM EDTA, 20 mM Tris (pH 8.1), and 500 mM NaCl], LiCl wash buffer [0.25 M LiCl, 1% NP-40, 1% deoxycholate, 1 mM EDTA and 10 mM Tris (pH 8.0)] and TE buffer. The bead complexes were eluted by adding elution buffer (1% SDS and 0.1 M NaHCO_3_), and the eluted supernatants were subjected to cross-link reversal by adding RNase and NaCl and incubating at 65°C for 5 h. The samples were precipitated in 100% ethanol at −20°C for 12 h, and then incubated with proteinase K at 45°C for 2 h. The DNA fragments were purified using QIAQuick spin columns (Qiagen) and analyzed by semi-quantitative PCR using the KOD-Plus kit (Toyobo). The primer sequences are listed in Table 2.

**TABLE 2:** PCR primer sequences for mRNA quantification and ChIP assay

PCR primer	Sequences (5'→ 3')
Mouse Del-1_F	CTTGGTAGCAGCCTGGCTTT
Mouse Del-1_R	GCCTTCTGGACACTCACAGG
Mouse p53_F [41]	CCCGAGTATCTGGAAGACAG
Mouse p53_R [41]	ATAGGTCGGCGGTTCATGCC
ChIP-Del-1_ p53RE1_F(−1228/−1204)	CCACCTGAGTGATGTGTCAAGAATC
ChIP-Del-1_p53 RE1_R(−1105/−1126)	TCTGCTCTCAGTGGAGAAAGGG
ChIP-Del-1_ p53RE2_F(−1742/−1718)	CATGTATTGTCACCCTCTCACTCAG
ChIP-Del-1_ p53RE2_R(−1622/−1666)	CCAAATGAGGGCAAGAGAGAGAAG
Actin_F	AGCCATGTACGTAGCCATCC
Actin_R	CTCTCAGCTGTGGTGGTGAA

### Real-time RT-PCR

Total RNA was isolated from cells using Trizol (Invitrogen). cDNAs were generated using the ImProm-II reverse transcriptase kit (Promega). The cDNAs were subjected to real-time PCR amplification using LightCycler 480 SYBR Green 1 Master Mix and a LightCycler 480 machine (Roche, Mannheim, Germany). The primer sequences are listed on Table 2. The following PCR cycle was used: 95°C for 15 min; 50 cycles of 30 s at 95°C, 30 s at 60°C, and 30 s at 72°C; and 95°C for 15 min). Melting curve analyses were performed to ensure that specific PCR products were generated. Data were analyzed using the comparative threshold (C_T_) method [40], and the levels of mRNA were expressed as the relative fold change.

### Western blotting

Cells were washed with ice-cold PBS and lysed in a 1% Triton X-100-containing lysis buffer. Lysates were separated on a 12% SDS-PAGE gel and transferred to PVDF membranes. Membranes were blocked and probed with antibody against p53 (DO-1, Santa Cruz) or β-actin (Cell Signaling). After washing, the blots were incubated with appropriate HRP-conjugated secondary antibodies (Cell Signaling) and developed with SuperSignal West Pico Chemiluminescent Substrate kit (Thermo Scientific, Pittsburgh, PA). The band intensity was analyzed using ImageJ software.

### Statistical analysis

Data were compared using Student's t test. *p* < 0.05 was considered significant.
